# Fresh Blood Imaging using variable TR and variable refocusing flip angle for non-contrast peripheral MR angiography at 3T: a 7-Minute iliac to calf station run-offs scan

**DOI:** 10.1186/1532-429X-18-S1-P348

**Published:** 2016-01-27

**Authors:** Xiangzhi Zhou, Mitsue Miyazaki

**Affiliations:** Toshiba Medical Research Institute USA, Vernon Hills, IL USA

## Background

Non-contrast Fresh Blood Imaging (FBI) scan time can be greatly reduced using variable TR for imaging the peripheral artery without contrast infusion, in which each slice encoding (SE) and the followed echo train are played in a variable TR (vTR) (TR=n*RR) pattern. In vTR FBI, the lower limit of TR depends on the length and timing of data acquisition, and it is also limited by SAR, which is mostly affected by refocusing flip angle, echo train length, and echo spacing. Often in the case of short TR FSE acquisition, SAR can be an issue for the patient with fast heart rate. To reduce SAR so that the shortest TR can be enabled for the slice encodings at the kz edge, variable refocusing flip angle (vFA) is proposed in this work. The optimized vTR FBI sequence with vFA was applied on volunteers to achieve a 7-minutes 3 station run-off scan.

## Methods

The study was approved by our institutional review board and informed consent was obtained. Four volunteers were enrolled and scanned by a Vantage Titan 3T scanner (Toshiba Medical Systems Corporation, Otawara, Japan) equipped with Atlas SPEEDER Spine and Body coil. Followed by the localizer, pelvic, thigh and calf stations were imaged using the proposed FBI sequence with ECG gating. The FBI sequence is modified to incorporate the vTR and vFA functions, i.e., the slice encoding steps at the k-space center have longer TR (increased number of RR intervals) and higher refocusing flip angle and the number of the slice encoding steps with longer TR and/or higher refocusing flip angle can be adjusted. FBI parameters: 3D coronal single shot fast spin echo with half Fourier in PE direction, TR = 2RR with vTR (1 extra RRs for the middle 20% SE steps), TE = 60 ms, 50-80 slices for each station, slice thickness = 3 mm, matrix 256 × 256; FOV 37 cm × 37 cm, parallel imaging factor = 2, refocusing flip angle = 140° (160° the middle 20% SE steps), the acquisition delay times were determined by DelayTracker; resolution 1.4 mm × 1.4 mm, refined in the RO, PE and SE directions.

## Results

**Figure 1:**
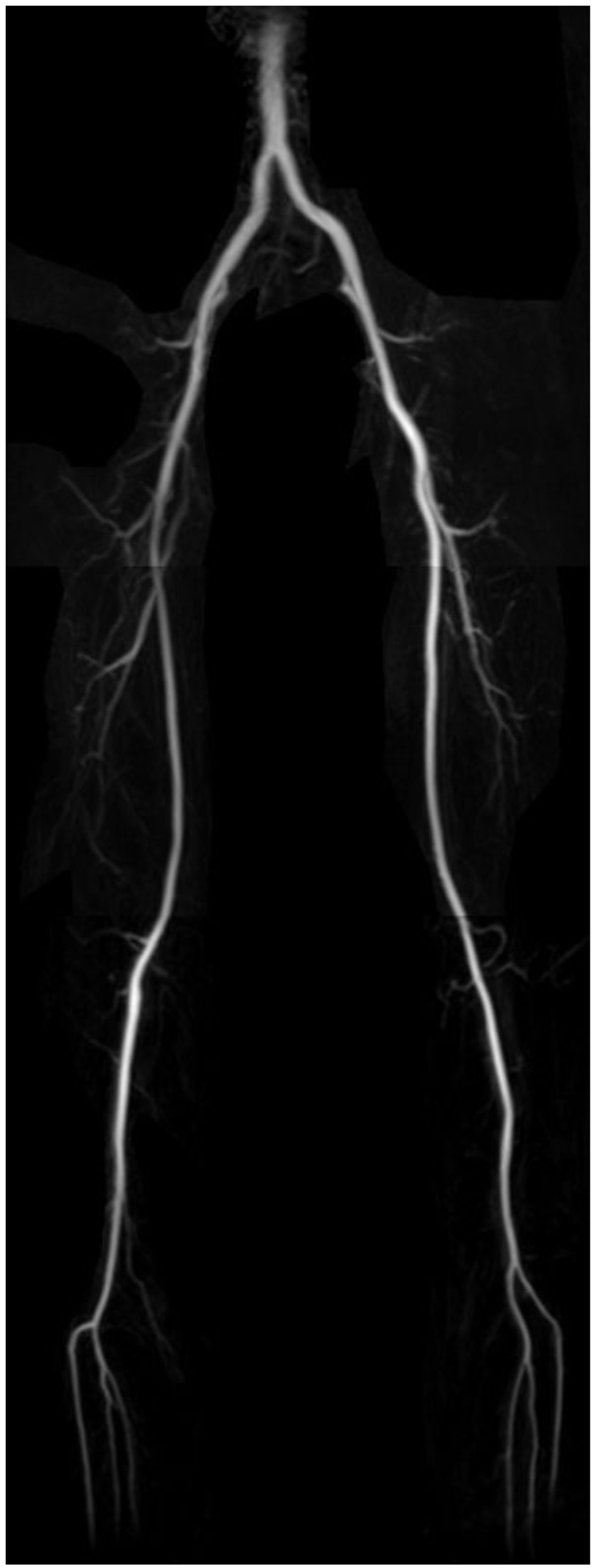
**Three station MIP image of a volunteer using FBI with vTR and vFA functions**. The total scan time is ~7 mins.

Fixed TR (TR = 3RR) FBI and vTR (TR = 2RR+20%3RR) FBI with vFA coronal MIP images at the 3 stations showed comparable arterial image quality across all volunteers and the scored MIP image quality has no significant difference. For the main arteries and smaller branches, vFA has the ability depicting both main arteries with higher refocusing flip angle and smaller vessels with lower refocusing flip angle (Figure 1). In the volunteer with narrower lumen on iliac arteries, FBI MIP images with and without vTR function can both clearly delineate the narrower lumen at different sites.

## Conclusions

The proposed vTR method with vFA offers significant scan time and SAR reductions for the FBI scan. Compared to fixed TR FBI, vTR can shorten the total scan time by 20-40%, and vFA can reduce the SAR for the echo train inside the short TR. This study demonstrated 7-mins 3 station peripheral MRA is feasible using the improved FBI technique. More data will be collected to further evaluate vTR FBI with vFA at 3T.

